# Efficacy of afoxolaner (NexGard®) against natural infestations with *Trichodectes canis* in dogs under field conditions

**DOI:** 10.1186/s13071-022-05428-y

**Published:** 2022-09-07

**Authors:** Andrei Daniel Mihalca, Georgiana Deak, Luciana Cătălina Panait, Ștefan Rabei, Frederic Beugnet

**Affiliations:** 1 Department of Parasitology and Parasitic Diseases, University of Agricultural Sciences and Veterinary Medicine of Cluj-Napoca, Calea Mănăștur 3-5, 400372 Cluj-Napoca, Romania; 2 Parasitology Consultancy Group SRL, Strada Principală 145B, 407056 Corușu, Romania; 3 Boehringer Ingelheim Animal Health, 29 Av. Tony Garnier, 69007 Lyon, France

**Keywords:** Afoxolaner, Chewing lice, Efficacy assessment, *Trichodectes canis*

## Abstract

**Background:**

*Trichodectes canis* is a small chewing louse found globally that primarily infests dogs. Limited information is available on the efficacy of isoxazolines against infestation with the chewing louse. In the present study, we evaluated the efficacy of afoxolaner, an isoxazoline class compound, in naturally infested domestic dogs.

**Methods:**

The field study was carried out in Romania. Between September 2021 and December 2021, 43 dogs with confirmed *T. canis* infestation were included in the study. On the day of the inclusion (day 0), each animal was clinically examined and randomly treated with a control product labeled for use against lice [fipronil-(S)-methoprene combination (Frontline Combo®; Boehringer Ingelheim)] or with the investigational product [chewable tablets containing afoxolaner (NexGard®; isoxazoline)]. Each animal was evaluated for the presence of lice at 15 and 30 days post-inclusion.

**Results:**

Of the 48 dogs initially included in the study, 43 completed the treatment period [18 in the control group (CG) and 25 in the investigational group (IG)]. At day 14, no living *T. canis* lice were detected on the dogs in either group. At day 14, dead lice were detected in four dogs in the IG, while eggs were present in two dogs in the IG and in one dog in the CG. At day 30, no lice were detected in either group, while eggs were still present in one dog in the CG.

**Conclusion:**

These results suggest that afoxolaner is a feasible treatment option against chewing lice in dogs, providing 100% curative efficacy.

**Graphical Abstract:**

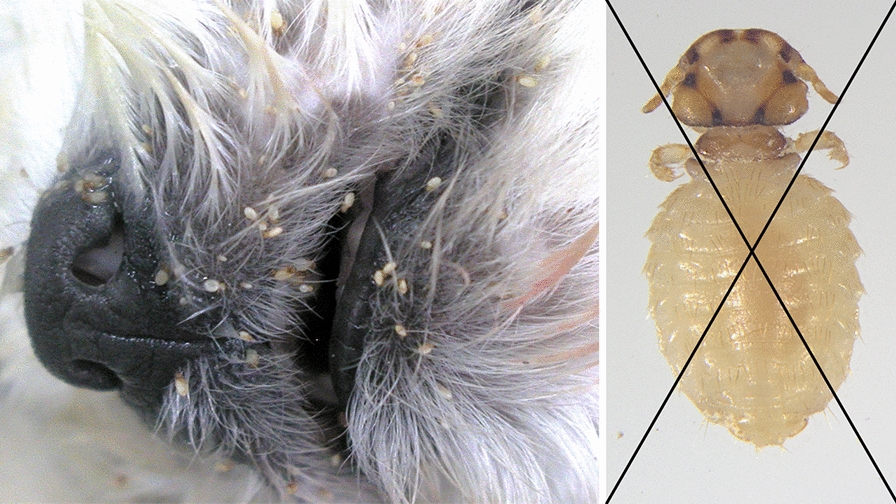

**Supplementary Information:**

The online version contains supplementary material available at 10.1186/s13071-022-05428-y.

## Background

*Trichodectes canis* (Phthiraptera, Mallophaga, Trichodectidae) is a species of chewing louse distributed worldwide. It primarily infests domestic dogs, but has also been reported to infest wild canids and other wild carnivores [1, 2]. This louse is host-specific and, consequently, there is no risk of transmission to other domestic species, such as cats, or to humans [3]. General consensus is that owned dogs are rarely infested with *T. canis* and that infestation is more common in stray animals [3], with the prevalence of *T. canis* infestation varying greatly by country and dog category, from 0.2% up to 10.6% [4–10].

Small numbers of lice are generally not associated with clinical signs in dogs, but heavy infestations can cause hair loss, pruritus and scales [3]. The diagnosis of lice infestation is based on finding active life-cycle stages (i.e. nymphs and adults) on the body surface of infested dogs or finding nits attached to their hairs. Together with fleas, *T. canis* can also serve as an intermediate host for the zoonotic tapeworm *Dipylidium caninum* [3].

Due to the negative impact on dog health and welfare, as well as the risk of *D. caninum* transmission, all dogs confirmed to be infested with *T. canis* must be treated. To date, several clinical studies have evaluated the efficacy of insecticides against *T. canis* in dogs [11–17]. The results of these studies show that insecticides such as propoxur, fipronil, imidacloprid, selamectin and pyrethroids are effective after a single topical administration of different formulations (i.e. collars, spots-on or sprays) [11–17]. Isoxazoline insecticides have been available on the veterinary pharmaceutical market for almost a decade, but their efficacy against *T. canis* has not yet been evaluated.

Oral chewable formulations of afoxolaner and fluralaner were the first isoxazoline insecticides to be marketed (in 2013–2014), followed by sarolaner in 2015 and lotilaner in 2017 [18]. These insecticides act through inhibition of the helical subunits of gamma-aminobutyric acid (GABA), a neurotransmitter found in the peripheral nervous system of invertebrates, and have a strong inhibitory activity on the glutamate-gated chloride channel in invertebrates [19, 20]. They are considered to be safe [21, 22] and broad-spectrum ectoparasiticides for pets, with a demonstrated activity against several species of ticks, *Demodex*, *Sarcoptes*, *Otodectes*, fleas and sucking lice of dogs and cats [20, 23]. Isoxazolines have a systemic mode of action as they are highly bound on plasma proteins [23, 24], and are ingested by hematophagous arthropods (fleas and ticks) during their blood meal. The demonstrated efficacy against non-strictly hematophagous arthropods, such as mites, may be related to their presence in inflammatory products containing plasma proteins [26–30]. To date, no resistance against isoxazolines has been reported.

As chewing lice are superficial ectoparasites, considered to induce a very moderate skin inflammatory reaction, we thought it important to assess the potential efficacy of a systemic molecule administered orally. The aim of the study was therefore to determine the efficacy of a single dose of the oral formulation of afoxolaner (NexGard®; Boehringer Ingelheim, Ingelheim am Rhein, Germany) for the treatment of naturally acquired chewing lice (*Trichodectes canis*) infestation in dogs under field conditions, and to compare this efficacy to that of a topical ectoparasiticide acting by contact and registered in Europe for its efficacy against dog chewing lice [i.e. fipronil-(S)-methoprene] (Frontline Combo®; Boehringer Ingelheim).

## Methods

### Study site and included animals

This was a multi-site, positive-control, blinded clinical efficacy field study that was implemented in the historical region of Transylvania, Romania. Between 17 September and 4 December 2021, we included 43 dogs [24 females (2 neutered), 19 males (1 neutered)] aged between 2 months and 20 years (31 dogs aged < 6 months; 4 dogs aged 6–12 months; 1 dog aged 12–24 months; 7 dogs aged > 24 months) in the study. The dogs originated from six counties (16 localities), as follows: Bihor (Oradea: 1 dog), Bistrița-Năsăud (Beclean: 3 dogs; Dumbrava: 1 dog; Enciu: 5 dogs; Feleac: 2 dogs; Malin: 2 dogs; Nușeni: 9 dogs; Rusu de Jos: 2 dogs; Rusu de Sus: 2 dogs; Vișa: 2 dogs), Cluj (Cămărașu: 1 dog; Corușu: 7 dogs; Popești: 1 dog), Hunedoara (Hunedoara: 2 dogs), Sibiu (Săcădate: 1 dog) and Suceava (Solca: 2 dogs). Details of the dogs included in the study are shown in the Additional file 1: Table S1. Five dogs initially included in the study were removed from the analysis as they were lost to follow-up (*n* = 3), died from unknown cause (*n* = 1) or changed their owner during the study (*n* = 1).

### Inclusion, exclusion and removal criteria

For inclusion, the body surface of privately owned and shelter dogs was carefully inspected for the presence of the chewing louse *T. canis*. Only dogs found to be infested with *T. canis* (based on the presence of motile adult stages and at least 1 nit), clinically healthy (with the exception of skin lesions consistent with mallophagosis, such as pruritus, hair loss and presence of scales), weighing at least 2 kg and aged at least 8 weeks were included in the study. The animals had not been treated with any ectoparasiticide within the previous 3 months. The aim of the study was explained to the owners, who were asked to sign an informed consent form.

For each animal included in the study, after visual confirmation of the presence of lice, one adult louse was collected in absolute ethanol and later confirmed microscopically as *T. canis*, according to standard morphological criteria [31].

The exclusion criteria were: (i) presence of clinical signs other than those consistent with the presence of chewing lice; (ii) treatment with topical or systemic ectoparasiticides within the last 3 months or within the efficacy duration of the respective ectoparasiticide drug; (iii) pregnant or lactating females; and (iv) females intended for breeding during the study period. Any dog meeting the exclusion criteria were not included in the study even if they met the inclusion criteria.

After inclusion, dogs were excluded if they subsequently fell into any of the removal criteria categories: death, loss or disappearance of dog; change of owner; withdrawal of owner consent; inappropriate health status or behavior of the dog in the context of the study; and dogs from sites that had been treated with environmental ectoparasiticides after inclusion in the study and before completion. All dogs remained in their respective households during the investigation period.

### Randomization, study groups treatment and evaluation

After inclusion in the study, each dog was randomly assigned to one of the two study groups. Dogs in group 1, the investigational group (IG), were treated with NexGard® according to the dosing table on the product label), and dogs in group 2, the positive control group (CG), were treated with Frontline Combo® according to the label instructions. If several dogs from the same household/owner or shelter were included in the study, they were all allocated to the same study group. If other dogs than the one(s) included were present in the same household or shelter, they were all treated with the same product as the included dog(s), even in the absence of chewing lice infestation.

Clinical evaluations were performed at days 0 (inclusion), 14 and 30 and consisted of scoring and recording the skin lesions and symptoms, as follows: pruritus (0 = absent; 1 = mild without alteration of the skin; 2 = moderate with mild alterations of the skin; 3 = severe with pronounced alterations of the skin), hair loss (0 = absent; 1 = very limited; 2 = mild; 3 = extensive); and presence of scales (0 = absent; 1 = very limited; 2 = mild; 3 = extensive). Additionally, a scoring system was applied for grading the presence of chewing lice, as follows: 1 (nits + 1 adult chewing louse); 2 (nits +  < 10 chewing lice); or 3 (nits +  > 10 chewing lice). No distinction was made between nymphs and adults during the lice count. The presence of other ectoparasites (fleas, hard ticks) was also recorded. No skin scrapings were done when lesions were visible.

### Statistical analysis

Statistical associations between ordinal data, such as days 0, 14 and 30 and the scores used to assess pruritus, hair loss and the presence of scales and lice, were assessed using the non-parametric Wilcoxon signed-rank test. A *P *value < 0.05 was considered to be statistically significant. Data were analyzed using R software v. 4.0.5 (R Foundation for Statistical Computing, Vienna, Austria).

## Results

Of the 43 dogs included in this study, 25 were assigned to group 1 (IG, treated with NexGard®) and 18 were assigned to group 2 (CG, treated with Frontline Combo®). The score for lice infestation on day 0 was 2 or 3 for all dogs (Table 1). On day 14, all dogs from both groups scored 0 for lice (i.e. no living lice detected). On day 14, of the 25 dogs in group 1, two still had nits and four had dead lice, and of the 18 dogs in group 2, nits were found on one dog. Similarly, on day 30, all dogs from both groups scored 0 for the presence of live lice; no dead lice or nits were found on dogs in group 1, while one dog in group 2 still had nits. During the 30 days of surveillance, no reinfestations due to the hatching of eggs were observed. These results demonstrate a clinical efficacy of 100% for the oral formulation of afoxolaner (NexGard®). Moreover, during the evaluation period, none of the dogs suffered any adverse reactions.

**Table 1:** Scoring of lice infestation at inclusion in the 43 dogs that completed the study

Groups^a^	Lice infestation score 2 (*n*)^b^	Lice infestation score 3 (*n*)^b^	Total
Group 1	7	18	25
Group 2	9	9	18
Total	16	27	43

^a^Group 1 (investigational group) dogs were treated with NexGard® according to the dosing table on the product label); group 2 dogs (positive control group) were treated with Frontline Combo® according to the label instructions

^b^Presence of chewing lice was graded as follows: 1 (nits + 1 adult chewing louse); 2 (nits +  < 10 chewing lice);r 3 (nits +  > 10 chewing lice)

**Fig. 1:**
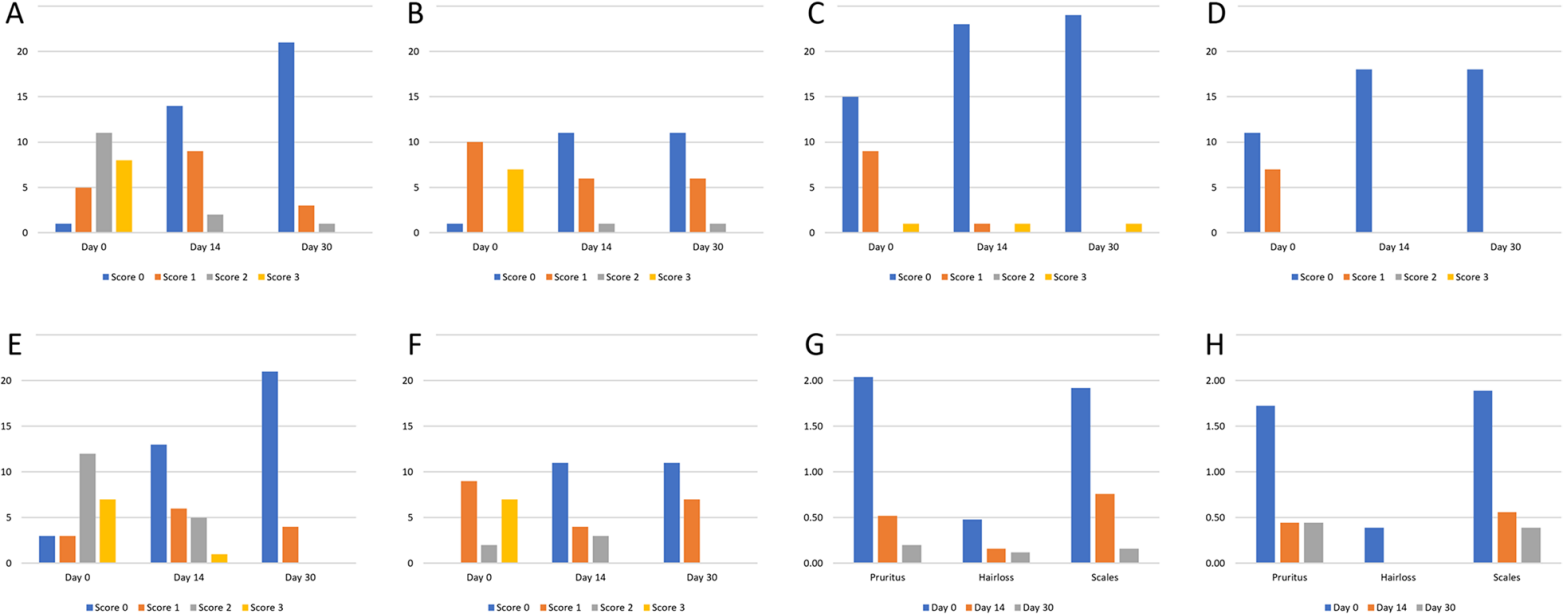
Clinical scores for all evaluated dermatological signs according to number of dogs (*Y*-axis). Group 1 (investigational group) dogs were treated with NexGard®, and group 2 (positive control group) dogs were treated with Frontline Combo®. **a**, **b** Clinical score for pruritus in group 1 dogs (**a**) and in group 2 dogs (**b**). **c**,** d** Clinical score for hair loss in group 1 dogs (**c**) and in group 2 dogs (**d**). **e**,** f** Clinical score for scales in group 1 dogs (**e**) and in group 2 dogs (**g**). **g**,** h** Average score for the clinical signs associated with lice in group 1 dogs (**g**) and in group 2 dogs (**h**). See section Randomization, study groups treatment and evaluation for explanation of grading/scores

The clinical score for all evaluated dermatological signs improved in both groups on days 14 and 30 compared to day 0 (Fig. 1a–h). Following treatment, a statistically significant decrease in degree of pruritus, hair loss and presence of scales and in lice scores was observed in both groups at days 14 and 30 compared to day 0 (*P* < 0.001). No significant difference was observed between the two treatment groups. On day 0 (inclusion) 23 of the 43 dogs which completed the study were also infested with other ectoparasites (i.e. fleas and/or ticks which were not collected or identified to species level) (Table 2).

**Table 2:** Number of dogs infested by other ectoparasites in the two study groups

Group	Day 0	Fleas (*n*)	Ticks (*n*)	Fleas and ticks (*n*)
Group 1	0	10	0	0
	14	0	0	0
	30	0	0	0
Group 2	0	10	0	3
	14	0	0	0
	30	0	1	0
Total	0	20	0	3
	14	0	0	0
	30	0	1	0

Day 0 is day of inclusion in study; days 14 and 30 are time points of evaluation post treatment

## Discussion

Modern veterinary practitioners desiderate antiparasitic drugs due to their broader spectrum, higher efficacy and lower toxicity both for the animals and for the environment. However, due to their more recent availability on the market, their full spectrum is not fully known due to the lack of studies.

Several studies have evaluated the field efficacy of various ectoparasiticides against chewing lice infestation in dogs. These are summarized in Table 3. All studies included a single dose of the test drug and reported a 100% efficacy as early as 7 days post-treatment. In addition, one study reported a 100% efficacy of the tested concentrate, which is extracted from neem tree seeds [32].

**Table 3:** Overview of the efficacy field studies of various antiparasitic products against *Trichodectes canis*

Molecule(s)	Formulation	Days	Efficacy	Reference
Propoxur	Collar	2, 28, 42	98.5–100%^a^	[14]
Imidacloprid	Spot-on	1, 14, 28, 42	100%^b^	[11]
Imidacloprid + Flumethrin	Collar	2, 7, 14, 21, 28, 35	95.1–100%^c^	[16]
Permethrin	Spot-on	7, 14, 21, and 28	100%	[13]
Fipronil	Spot-on	2, 7, 14, 21, 28, 35	100%^d^	[18]
Fipronil	Spot-on	2, 28, 42	98.3–100%^e^	[14]
Fipronil	Spray	2, 28, 42	99.6–100%^f^	[14]
Fipronil	Spray	2	100%	[10]
Selamectin	Spot-on	7, 14, 21, 28, 35, 42	100%	[15]
Selamectin	Spot-on	7, 14, 21, 28, 35, 42	100%	[35]
Afoxolaner	Oral	14, 30	100%	Current study

^a^98.5% efficacy on day 2, 100% efficacy on days 28 and 42

^b^Some dogs were also infested with the sucking louse *Linognathus setosus*; efficacy values are not given for day 1

^c^95.1% efficacy on day 2, 100% efficacy from day 7 onwards

^d^Live lice were still present on day 2

^e^98.3% efficacy on day 2, 100% efficacy on days 28 and 42 data and the results of the periodical checking

The present study is the first to evaluate the efficacy of an isoxazoline, afoxolaner, against *T. canis* in dogs and the first to evaluate the efficacy of afoxolaner against mammalian lice. Afoxolaner has previously been evaluated in off-label clinical studies against the chewing louse *Goniodes pavonis* in captive aviary birds, where it demonstrated a 100% efficacy at 28 days post-treatment in various species of pheasants [33] and an 86.6% efficacy in peacocks [34]. Kohler-Aanesen et al. [23] reported an efficacy of 85.1% on day 1, 96.8% on day 7 and 100% on days 28 and 84 for an oral formulation of fluralaner against the dog sucking louse *Linognathus setosus*.

Our study results confirm that afoxolaner, despite a systemic distribution and mode of action [24, 25], is able to kill superficial chewing lice when administered orally. It could be hypothesized that even when clinically limited, the inflammatory process during chewing lice infestation is sufficient to enable afoxolaner to penetrate into the epidermis in a concentration that allows the killing of *Trichodectes canis*.

### Conclusion

In conclusion, afoxolaner showed a 100% efficacy for the treatment of infestation with the canine chewing louse *T. canis*, adding another canine ectoparasite to its already well-known broad spectrum.

## Supplementary Information

**Additional file 1:** **Table S1.** Dataset representing all the collected data and the results of the periodical checking’s.

## Data Availability

The dataset analyzed in the current study is available in the Additional file 1: Table S1. The lice specimens collected are available from author FB on reasonable request.
